# Small RNAs from the plant pathogenic fungus *Sclerotinia sclerotiorum* highlight host candidate genes associated with quantitative disease resistance

**DOI:** 10.1111/mpp.12841

**Published:** 2019-07-30

**Authors:** Mark Derbyshire, Malick Mbengue, Marielle Barascud, Olivier Navaud, Sylvain Raffaele

**Affiliations:** ^1^ Centre for Crop and Disease Management Curtin University Perth Western Australia Australia; ^2^ Laboratoire des Interactions Plantes Micro‐organismes INRA, CNRS, Université de Toulouse Castanet Tolosan France

**Keywords:** *Arabidopsis*, effector, GWAS, necrotrophic fungus, plant immunity, plant pathogen, RNAi

## Abstract

Fungal plant pathogens secrete effector proteins and metabolites to cause disease. Additionally, some species transfer small RNAs (sRNAs) into plant cells to silence host mRNAs through complementary base pairing and suppress plant immunity. The fungus *Sclerotinia sclerotiorum* infects over 600 plant species, but little is known about the molecular processes that govern interactions with its many hosts. In particular, evidence for the production of sRNAs by *S. sclerotiorum* during infection is lacking. We sequenced sRNAs produced by *S. sclerotiorum in vitro* and during infection of two host species, *Arabidopsis thaliana* and *Phaseolus vulgaris*. We found that *S. sclerotiorum* produces at least 374 distinct highly abundant sRNAs during infection, mostly originating from repeat‐rich plastic genomic regions. We predicted the targets of these sRNAs in *A. thaliana* and found that these genes were significantly more down‐regulated during infection than the rest of the genome. Predicted targets of *S. sclerotiorum* sRNAs in *A. thaliana* were enriched for functional domains associated with plant immunity and were more strongly associated with quantitative disease resistance in a genome‐wide association study (GWAS) than the rest of the genome. Mutants in *A. thaliana* predicted sRNA target genes *SERK2* and *SNAK2* were more susceptible to *S. sclerotiorum* than wild‐type, suggesting that *S. sclerotiorum* sRNAs may contribute to the silencing of immune components in plants. The prediction of fungal sRNA targets in plant genomes can be combined with other global approaches, such as GWAS, to assist in the identification of plant genes involved in quantitative disease resistance.

## Introduction

Fungal phytopathogens largely rely on small secreted proteins, termed effectors, to infect and cause disease (Lo Presti *et al.*, 2015). Effectors can enter plant cells or act in the apoplast to manipulate host cell functions and promote fungal invasive growth (Toruño *et al.*, 2016). Necrotrophic fungi, which actively kill host cells, may also secrete various metabolites to facilitate plant colonization (Friesen *et al.*, 2008). Recently, small RNAs (sRNAs) produced by the plant pathogenic fungus *Botrytis cinerea* were also shown to be transferred into host tissues, where they silence host genes to facilitate infection (Weiberg *et al.*, 2013). Subsequent studies have characterized a further sRNA from *B. cinerea* and sRNAs from the wheat rust fungus *Puccinia striiformis* f. sp. *tritici* with host‐gene silencing properties (Mueth *et al.*, 2015; Wang *et al.*, 2016).

sRNAs are short non‐coding sequences of RNA that are usually between 20 and 30 nucleotides long (Dang *et al.*, 2011). Complementary base pairing of sRNAs with mRNA sequences guides a group of proteins, called the RNA‐induced silencing complex, that mediate mRNA degradation or inhibition of translation (Pratt and MacRae, 2009). This process is known as RNA silencing, and it is involved in various cell functions such as development, transcription, translation and defence against viruses and transposable elements (Dang *et al.*, 2011). In *B. cinerea,* a single sRNA potentially has the ability to target 15 genes in *Arabidopsis thaliana*, including WRKY transcription factors, receptor‐like kinases and cell wall‐modifying enzymes (Wang *et al.*, 2016). Many of the host genes targeted by the sRNAs of pathogenic fungi discovered to date exhibit functional domains typically associated with plant immune responses. In this way, fungal pathogen sRNA function may be analogous to that of pathogen effector proteins.

Another feature of sRNAs that makes them similar to effectors is their association with repetitive sequences. Many sRNAs are, in fact, directly transcribed from transposable elements (Dang *et al.*, 2011). The genomes of several filamentous pathogens have evolved towards compartmentalization into repeat‐rich, gene‐sparse regions that contain effector genes, and repeat‐poor gene‐rich regions that contain housekeeping genes (Dong *et al.*, 2015; Fouché *et al.*, 2018). Although there have been several sRNA profiling studies on plant pathogenic fungi (Chen *et al.*, 2014; Mueth *et al.*, 2015; Zhou *et al.*, 2012), whether sRNA loci are associated with repeat‐rich, gene‐poor regions of fungal genomes has not yet been considered.

Plant resistance to several fungal pathogens relies on the recognition of a single fungal effector by a plant resistance protein in a ‘gene‐for‐gene’ manner (Fenton *et al.*, 2009; Jones and Dangl, 2006). This leads to simple segregation between fully susceptible and fully resistant plants in host populations. However, infection by *Sclerotinia sclerotiorum* results in a gradient of resistance phenotypes, controlled by a complex genetic programme designated as quantitative disease resistance (QDR) (Mbengue *et al.*, 2016; Roux *et al.*, 2014).

Because QDR relies on numerous small‐effect loci, unravelling the molecular basis of QDR is a major challenge in plant pathology (Peyraud *et al.*, 2017; Poland *et al.*, 2009; Roux *et al.*, 2014). To identify portions of the genome containing markers linked with the QDR response, studies have used association genetics approaches (Bergelson and Roux, 2010; Poland *et al.*, 2011) such as genome‐wide association studies (GWASs) (Brachi *et al.*, 2011). In the context of plant disease resistance, GWASs involve subjecting a diverse group of natural plant accessions to the same disease pressure. Linear models are then used to assess the predictive power of genomic markers for the level of disease whilst accounting for population structure (Bradbury *et al.*, 2007; Purcell *et al.*, 2007; Zhang *et al.*, 2010). However, GWASs are limited by their ability to detect rare alleles and identify causative mutations associated with traits controlled by a large number of small effect loci (Bergelson and Roux, 2010). To circumvent these limitations, GWASs can be combined with other approaches such as biparental quantitative trait locus (QTL) mapping (Huard‐Chauveau *et al.*, 2013) and RNA sequencing (Badet *et al*., 2017a; Chan *et al.*, 2011). Since sRNAs from plant pathogenic fungi are likely to suppress host genes functioning in disease resistance, we hypothesized that the prediction of plant genes targeted by fungal sRNAs could be combined with a GWAS to aid identification of plant genes relevant to QDR.

Like *B. cinerea*, *S. sclerotiorum* is a plant pathogen belonging to the Sclerotiniaceae family of Ascomycete fungi, which is able to infect hundreds of plant species (Kabbage *et al.*, 2015; Navaud *et al.*, 2018). *Sclerotinia sclerotiorum* is widely dispersed throughout the world and poses a significant threat to agricultural production (Derbyshire and Denton‐Giles, 2016). A finished genome for *S. sclerotiorum* strain 1980 is available (Derbyshire *et al.*, 2017) and GWAS of QDR to *S. sclerotiorum* in *A. thaliana* has been reported (Badet *et al*., 2017a). A previous study has shown that *S. sclerotiorum* produces sRNAs *in vitro* (Zhou *et al.*, 2012). Whether it also produces sRNAs during plant infection is, thus far, undescribed.

We found that *S. sclerotiorum* produces 374 highly abundant sRNAs during infection of two of its host species *Phaseolus vulgaris* and *A. thaliana*. In *A. thaliana*, predicted target genes were more likely to be significantly down‐regulated during infection than other genes. Among the *A. thaliana* targets, there were significantly more genes associated with QDR by GWAS than expected by chance. Our data indicate that plastic regions of the *S. sclerotiorum* genome generate transposable element‐derived sRNAs that potentially target numerous plant genes likely involved in QDR. They also suggest that fungal sRNA sequencing and identification of their plant targets can be complementary to other available approaches for the search of genes controlling plant QDR.

## Results

### *Sclerotinia sclerotiorum* sRNAs exhibit a characteristic length distribution and 5′ uridine bias

To identify *S. sclerotiorum* sRNAs expressed during infection of host plants, we conducted sRNA sequencing on *S. sclerotiorum* growing *in vitro* and during infection of the two host species, *A. thaliana* and *P. vulgaris*. All samples were collected in triplicate and both the centre and border regions of disease lesions were harvested *in planta*. After adapter trimming and quality filtering, we obtained a total of 112 835 544 *in planta* reads across 12 samples and 21 394 528 *in vitro* reads across three samples (Table S1). We filtered out reads that matched exactly (i) the sense strands of plant transcripts (either *P. vulgaris* or *A. thaliana*), (ii) plant non‐coding RNAs (either *P. vulgaris* or *A. thaliana*), (iii) plant sRNAs (either a mock‐inoculated *A. thaliana* sample or a *P. vulgaris* sRNA dataset obtained from Formey *et al.* (2015), (iv) the sense strands of *S. sclerotiorum* transcripts and (v) *S. sclerotiorum* non‐coding RNAs from Rfam. The *in vitro* samples were filtered twice, once using *A. thaliana* sequences for steps (i)–(iii) and once using *P. vulgaris* sequences for steps (i)–(iii) to ensure that samples had undergone the same filtering procedures for differential expression analysis. The filtering process resulted in 14 798 914 mapped reads from all *in planta* samples, 635 493 *in vitro* reads when filtered against *A. thaliana* and 756 907 *in vitro* reads when filtered against *P. vulgaris.*

**Figure 1:**
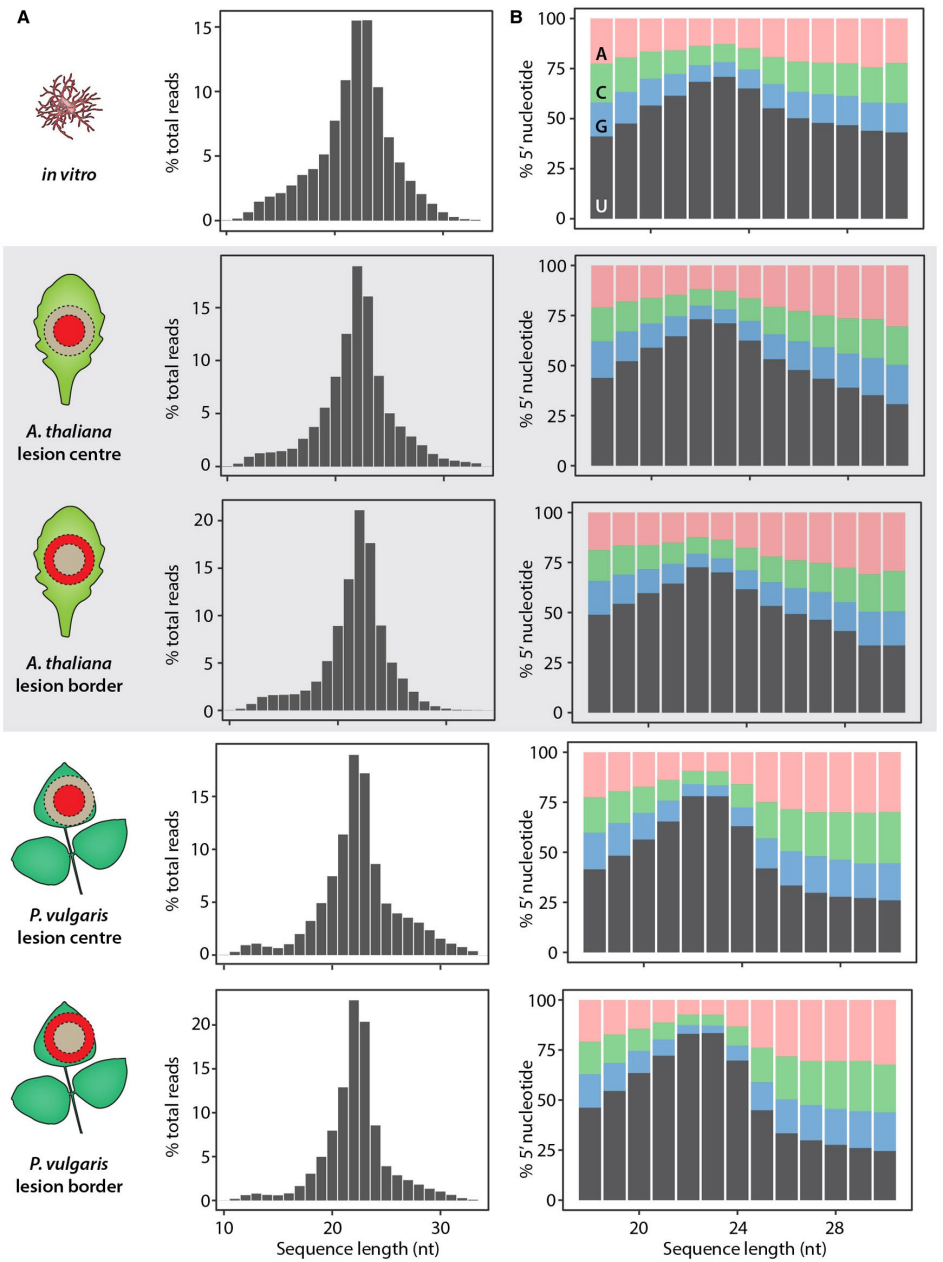
Length distribution and 5′ nucleotide bias of *Sclerotinia sclerotiorum* sRNAs. (A) The percentage of reads (*y*‐axis) according to nucleotide (nt) sequence length (*x*‐axis) obtained *in vitro,* in *Arabidopsis thaliana* lesion centres and borders, and in *Phaseolus vulgaris* lesion centres and borders; reads for this plot obtained before the specified filtering procedure. (B) The percentage of adenine (pink), cytosine (green), guanine (blue) and uridine (grey) in the 5′ position according to read length.

To determine the origin of sRNA reads in our samples, we analysed size distribution and 5′ nucleotide bias (Fig. 1). Nonspecific RNA degradation would result in uniform sRNA size distribution and random 5′ nucleotides (Mueth *et al.*, 2015). In contrast, >70% of raw reads were between 20 and 25 nucleotides long in all our samples. All *in planta* samples exhibited a peak in abundance at 22 nucleotides, whereas the *in vitro* sample exhibited equal proportions at 22 and 23 nucleotides (Fig. 1A). We also observed a bias toward uridine as the 5′ nucleotide in *S. sclerotiorum* sRNA reads (Fig. 1B). This bias was most pronounced for the 22 and 23 nucleotide reads in all samples. These data show that the sRNAs mapping to the *S. sclerotiorum* genome exhibited characteristics commonly attributed to sRNA biogenesis in diverse species.

### Numerous *S. sclerotiorum* sRNAs are highly expressed in two hosts

**Figure 2:**
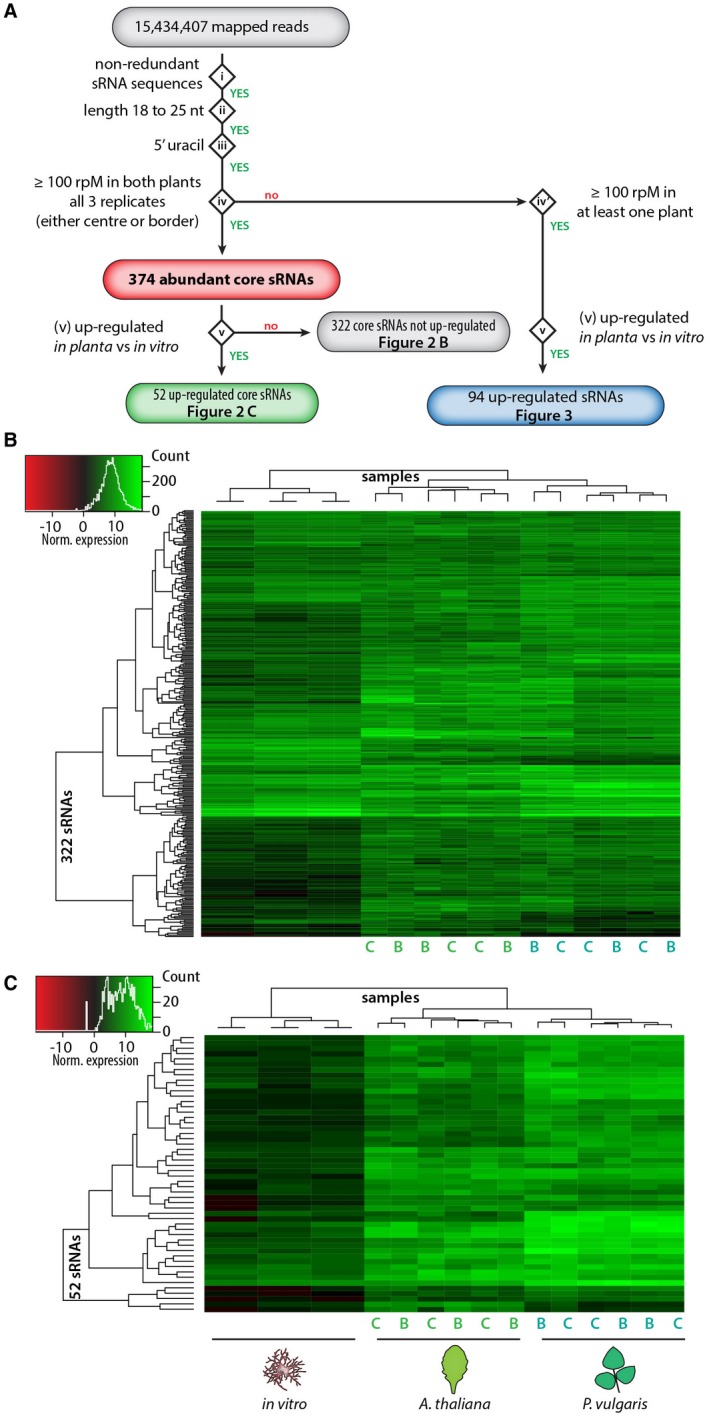
Identification of highly abundant fungal sRNAs and differential expression of fungal sRNAs *in planta*. (A) Five‐step (i to v) pipeline used to identify the 374 highly abundant core sRNAs in *Sclerotinia sclerotiorum* and differentially expressed sRNAs. Small RNAs expressed to a level of ≥ 100 reads per million (rpM) on both hosts in all replicates of at least one *in planta* sample (iv) were designated as abundant core sRNAs and were analysed for differential expression *in planta* relative to *in vitro* (v) (B, C). Heat maps of normalized expression data for the sRNAs identified using the procedure in (A). B, lesion border; C, lesion centre; FC, fold change; nt, nucleotides.

To identify *S. sclerotiorum* sRNAs that may be important for infection of host plants, we adapted proposed approaches for the identification of sRNA effector candidates (Weiberg *et al.*, 2013; Zanini *et al.*, 2018) and did not restrict the analysis to micro‐RNA generating loci. We focused on sRNAs matching all of the following criteria: (i) corresponding to non‐redundant sRNA sequences (i.e. all reads corresponding to a single sRNA sequence are counted together as 1), (ii) length between 18 and 26 nucleotides, (iii) harbouring a 5′ uridine and (iv) exhibiting over 100 reads per million *in planta*, in all three replicates collected from both host plants (either centre or border samples). This identified 374 abundantly expressed *S. sclerotiorum* core sRNA sequences in total (Fig. 2A, Data S1). By analysing the distribution of the most abundant size classes in read mapping loci in the genome (Mohorianu *et al.*, 2013) we were able to show that 369 of these sRNAs were derived from high confidence sRNA‐producing loci (Data S2). However, we kept all 374 sRNAs as there is currently relatively sparse knowledge of the nature of sRNA biogenesis in fungi.

Among these sRNAs, 322 were not significantly up‐regulated during infection (Fig. 2B), and 52 sRNAs (14%) were significantly up‐regulated during plant infection relative to *in vitro* (Fig. 2C). We did not find any sRNA reads that exhibited significant changes in abundance between the centres and borders of infection lesions (*P* adjusted > 0.05).

**Figure 3:**
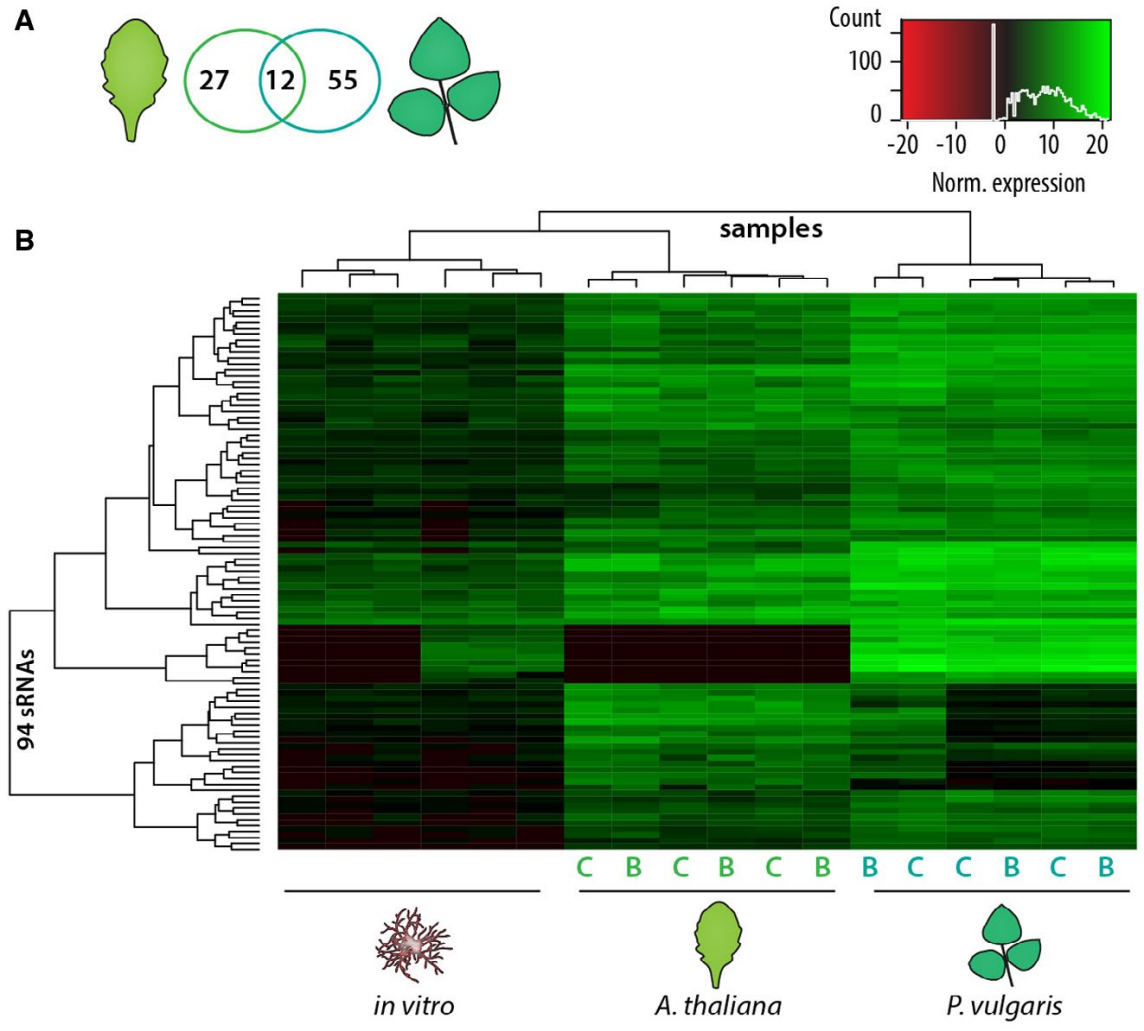
Differential expression of fungal sRNAs in *Arabidopsis thaliana* and *Phaseolus vulgaris*. (A) Venn diagram showing the number of *Sclerotinia sclerotiorum* sRNAs abundant (≥100 reads per million) and upregulated in *A. thaliana* or *P. vulgaris* relative to *in vitro*. (B) Heat map of normalized expression data for the 94 sRNAs identified in (A). B, lesion border; C, lesion centre; Norm., normalized; nt, nucleotides; rpM, reads per million.

To test whether the nature of the host plant affected the repertoire of sRNAs expressed by *S. sclerotiorum*, we considered sRNAs matching criteria (i)–(iii) above, showing over 100 reads per million *in planta* and significantly up‐regulated during infection of at least one host species (Fig. 2A). This identified a total of 94 sRNAs induced *in planta* (*P* adjusted < 0.05) (Fig. 3). Among these, 27 were up‐regulated on *A. thaliana* only, 55 were up‐regulated on *P. vulgaris* only and 12 were shared between both hosts.

Together, these data show that a number of sRNAs are highly abundant *in planta* and many are significantly up‐regulated during infection. Fungal cells residing at the centres and borders of disease lesions expressed similar sRNAs. In many cases, significant up‐regulation of sRNAs occurred specifically in one host. This conclusion should be taken with caution considering the lack of methods explicitly designed to quantify sRNA expression and the relatively low coverage in our *in vitro* samples that may bias differential expression analysis. Therefore, we focused the following analyses on the 374 sRNAs highly abundant during the infection of both *A. thaliana* and *P. vulgaris*.

### *Sclerotinia sclerotiorum* sRNAs map to transposable element sequences and are in gene‐poor polymorphic genome compartments

**Figure 4:**
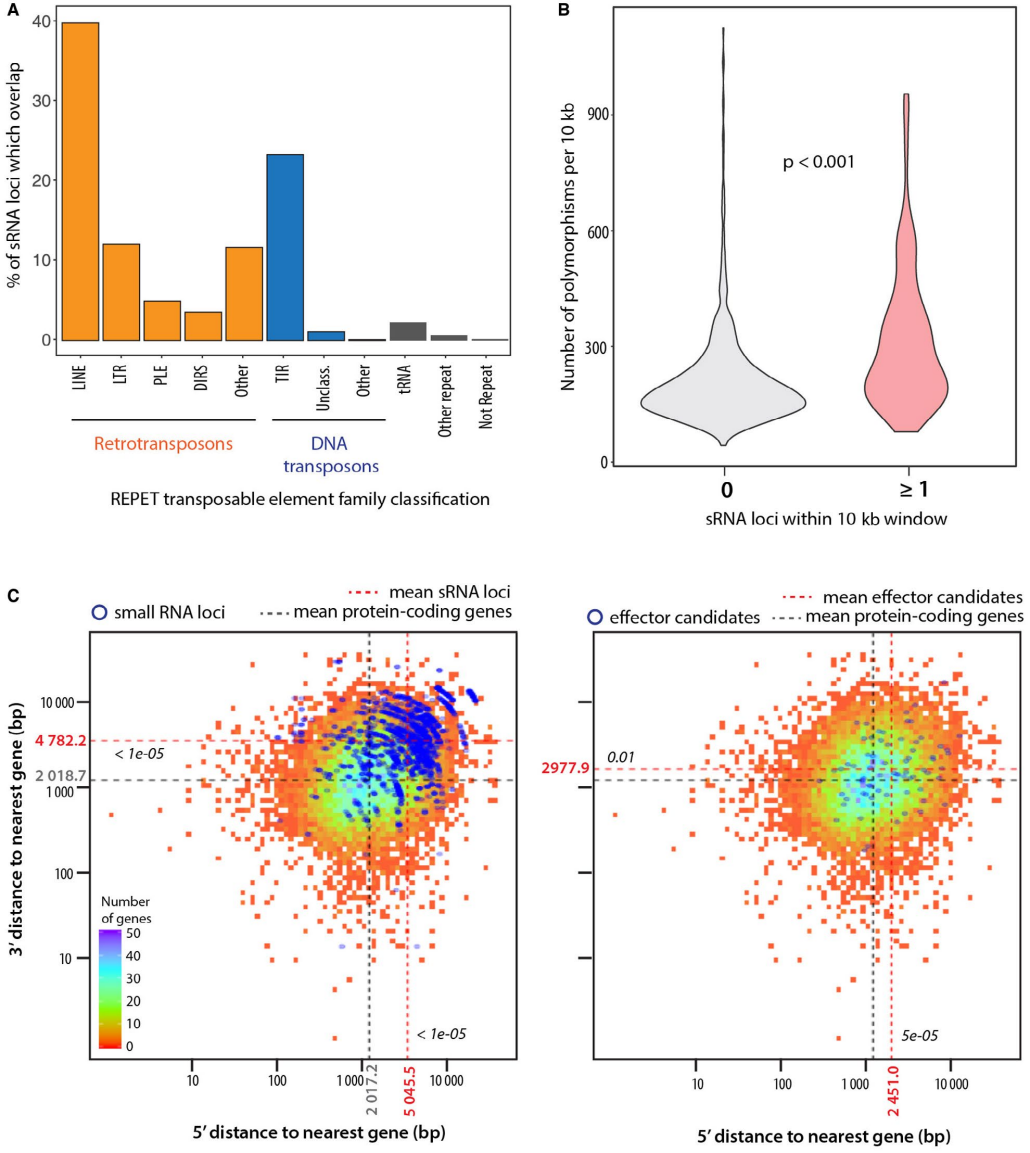
sRNA loci are associated with transposable elements, plastic and gene‐sparse genomic regions. (A) Percentage of sRNA loci (*y*‐axis) that overlap different classes of repeat sequence annotated by REPET (*x*‐axis). Unclass., unclassified. (B) Number of polymorphisms (*y*‐axis) in 25 *Sclerotinia sclerotiorum* isolates per 10 kb sliding window. Ten‐kb windows were analysed across the whole *S. sclerotiorum* genome and split into windows containing 0 and ≥ 1 sRNA locus (*x*‐axis). *P* value for a Wilcoxon’s rank sum test is shown. (C) Distance to neighbouring genes in base pairs (bp) from the 5′ (*x*‐axis) and 3′ (*y*‐axis) ends of sRNA loci (left) and effectors (right). Blue points represent sRNAs or effectors and the underlying heat map is for all *S. sclerotiorum* gene annotations. Mean distances for all *S. sclerotiorum* genes are show by grey dashed lines, mean distances for sRNAs or effectors are shown by red dashed lines represent with *P* value of a Wilcoxon’s test for significant difference.

To determine loci from which the 374 highly abundant sRNAs originated in the *S. sclerotiorum* genome, we analysed overlapping annotations for genomic regions they mapped to, with a particular interest in the previously published REPET analysis of transposable elements in *S. sclerotiorum* (Derbyshire *et al.*, 2017). Overall, 357 out of the 374 reads mapped to more than one place in the genome. We found that 3669 (99%) sRNA loci (including multiple mappings) were within 526 genomic regions annotated as transposable element sequence. The highest percentage of sRNA loci overlaps (32.5%) were with long interspersed nuclear elements (LINEs, Fig. 4A). To determine whether the sRNA loci were more polymorphic than other regions of the genome, we assessed the number of polymorphisms in 10 kb sliding windows throughout the genome from a panel of 25 *S. sclerotiorum* isolates (Derbyshire *et al.*, 2018). We counted polymorphisms if at least one individual did not exhibit the reference genome allele. The median number of polymorphisms was 235 for windows including at least one sRNA locus, but only 174 for windows not containing sRNA loci (*P* < 0.001, *W* = 54084, Wilcoxon’s rank sum test) (Fig. 4B). Strikingly, the proportion of windows showing 300 polymorphisms or more was 36% for windows including sRNA loci but only 12% otherwise.

Although the existence of repeat‐rich highly polymorphic genomic regions is well established in a number of filamentous plant pathogens (Dong *et al.*, 2015), the existence of such regions remains elusive in *S. sclerotiorum* (Derbyshire *et al.*, 2017). To determine the extent to which the sRNA loci reside in gene‐sparse regions, we calculated the distance between the 5′ and 3′ ends of all sRNA loci and the nearest gene borders, and for all *S. sclerotiorum* protein‐coding genes, including predicted effector genes from Derbyshire *et al. *(2017) (Fig. 4C). The mean distances to the nearest gene were 4898.56 bp (5′) and 4551.631 bp (3′) for sRNA loci, and only 2017.2 bp (5′, *P* < 2.2 × 10^−^^16^, *W* = 3053600) and 2018.7 bp (3′, *P* < 2.2 × 10^−^^16^, *W* = 31125000) for protein‐coding genes. The genes encoding effector candidates also resided further from the nearest gene than other protein‐coding genes (mean 2451.0 bp, *P* = 0.01005, *W* = 442360 and mean 2977.9 bp, *P* = 5.37 × 10^−^^05^, *W* = 480430 on 5′ and 3′ sides, respectively), albeit to a lesser extent than sRNA loci.

In addition to these analyses, we also focused on sRNA loci identified by the software package CoLIde. This program identifies the likely regions in the genome from which sRNAs may be derived based on read length bias and correlation in expression across samples. It aids detection of bona fide sRNA loci over false positives resulting from the inherently repetitive nature of sRNA mappings. We found that considering only the high‐confidence candidate sRNA loci from CoLIde did not strongly affect the conclusions about the gene‐sparseness and polymorphism of the genomic context of the 374 abundant sRNAs (Fig. S1). On average, CoLIde loci were further away from nearest neighbouring genes than genes were to each other (mean sRNA loci = 1782.371, mean genes = 1031.38, randomization p = 0) (Fig. S1A,B). Sliding windows containing CoLIde loci also contained significantly more polymorphisms than sliding windows that did not contain them (mean sRNA loci‐containing windows = 107.27, mean non‐sRNA loci‐containing windows = 54.9, Wilcoxon’s *P* = 2.2 × 10^−16^, *W* = 288550 (Fig. S1C). Together, these data indicate that *S. sclerotiorum* sRNAs are derived from gene‐sparse repetitive regions that are more polymorphic than the rest of the genome.

### Predicted targets of *S. sclerotiorum* sRNAs in *A. thaliana* are more likely to be down‐regulated during infection

**Figure 5:**
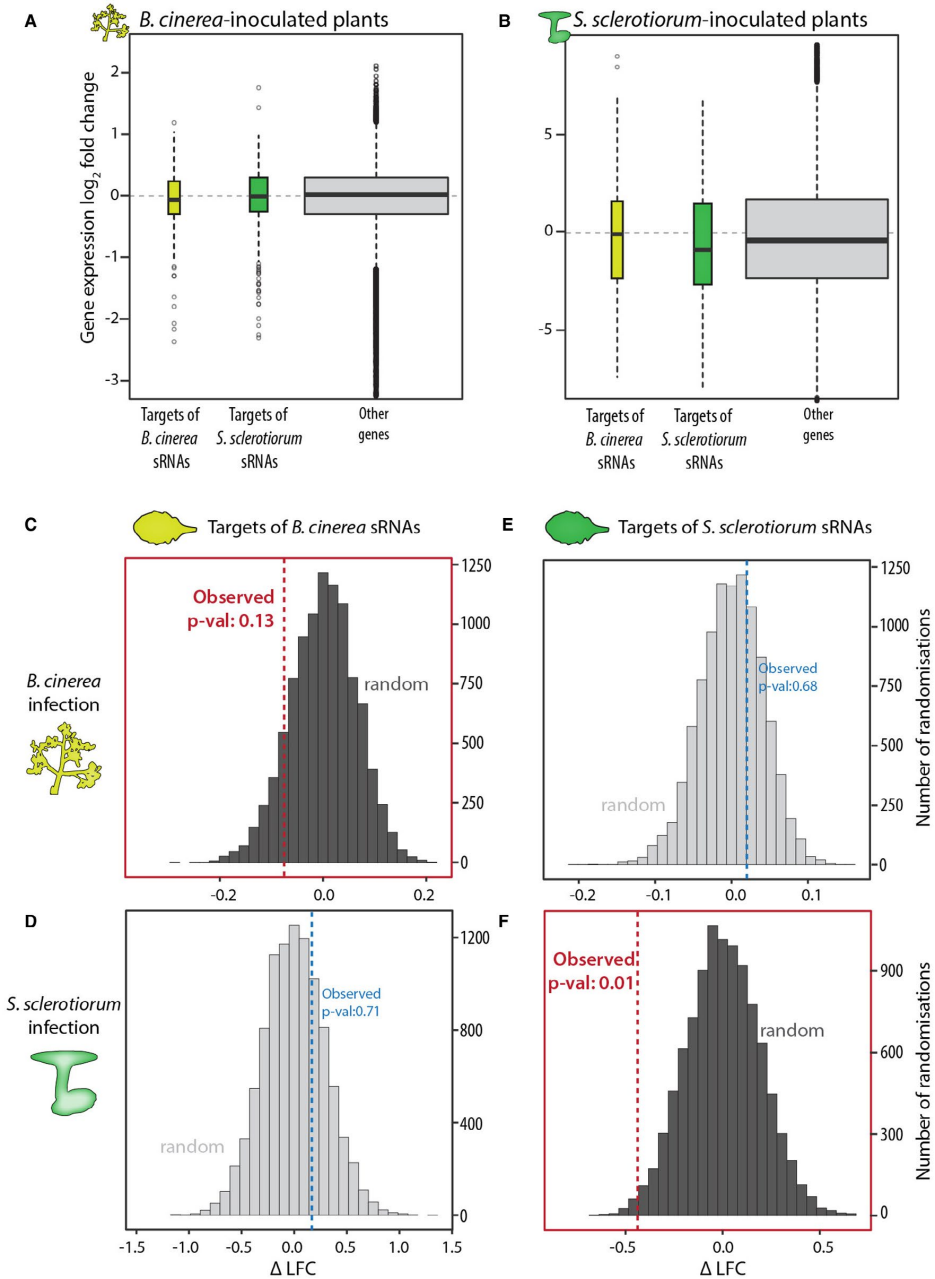
Predicted targets of *Sclerotinia sclerotiorum* sRNAs in *Arabidopsis thaliana* are significantly down‐regulated during infection. (A) Expression log_2_ (fold change) (LFC) (*y‐*axis) on inoculation by *Botrytis cinerea* for *A. thaliana* genes that were predicted targets of *B. cinerea* sRNAs (yellow), predicted targets of *S. sclerotiorum* sRNAs (green) and all other genes (grey)*.* Horizontal black lines represent median LFC, whiskers represent interquartile range and boxes represent second and third quartiles. (B) The same as for (A) on inoculation by *S. sclerotiorum*. (C)–(F) Distribution of difference in median LFC (∆LFC) between genes targeted or not by sRNAs in 10 000 randomizations (grey). Dashed vertical lines represent the observed ∆LFC. Predicted *A. thaliana* targets of *B. cinerea* sRNAs during infection with *B. cinerea* (C) and infection with *S. sclerotiorum* (D). Predicted *A. thaliana* targets of the 374 abundant *S. sclerotiorum* sRNAs during infection with *B. cinerea* (E) and infection with *S. sclerotiorum* (F).

Several ascomycete fungi produce sRNAs capable of modulating gene expression in their host plants (Wang *et al.*, 2016). To identify predicted targets of the 374 highly abundant *S. sclerotiorum* sRNAs in plant genomes, we used psRNATarget (Dai and Zhao, 2011). This server is designed to predict sRNA targets in plants. It first identifies sequences complementary to sRNAs in plant mRNAs using a Smith–Waterman alignment with a scoring schema based on known properties of plant miRNA + target interactions. The server also analyses target accessibility by determining the unpaired energy required to deconstruct the secondary structure around the sRNA target site (the UPE). To support host gene silencing by *S. sclerotiorum* sRNAs, we compared the expression of *A. thaliana* genes putatively targeted by fungal sRNAs with that of non‐target genes during fungal infection. We analysed predicted targets of *B. cinerea* sRNAs (Weiberg *et al.*, 2013), predicted targets of *S. sclerotiorum* sRNAs and all other *A. thaliana* genes during infection with *B. cinerea* (Coolen *et al.*, 2016) (Fig. 5A) or during infection with *S. sclerotiorum* (Fig. 5B). We considered gene expression log_2_(fold change) (LFC) relative to uninoculated controls. During *B. cinerea* infection, *A. thaliana* targets of *B. cinerea* sRNAs predicted by Weiberg *et al.* (2013) exhibited a lower median LFC (–0.064) than targets of *S. sclerotiorum* sRNAs (median LFC = –0.019) and other genes (median LFC = 0.013; Wilcoxon’s rank sum test *P* = 0.0989, *W* = 1862900). Compared to non‐target genes, the difference in median LFC (ΔLFC) was –0.077 for *B. cinerea* sRNAs targets and –0.032 for *S. sclerotiorum* sRNA targets. Conversely, during *S. sclerotiorum* infection, predicted *A. thaliana* targets of *S. sclerotiorum* sRNAs identified in this study exhibited a significantly lower LFC (–0.84) than targets of *B. cinerea* sRNA (median LFC = –0.14) and other genes (median LFC = –0.41; *P* = 0.03358, *W* = 4238200). Compared to non‐target genes, *S. sclerotiorum* sRNAs targets had a ΔLFC of –0.43 and *B. cinerea* sRNAs targets a ΔLFC of 0.27.

As a complementary analysis, we performed a randomization to test for the likelihood of finding a lower ΔLFC in random *A. thaliana* gene sets than observed for fungal sRNA targets. During *B. cinerea* infection, only 13% (equivalent to a one‐tailed *P* value of 0.13) of random *A. thaliana* gene samples had a lower ΔLFC than was observed from targets of *B. cinerea* sRNAs (Fig. 5C), but 71% had a lower ΔLFC than targets of *S. sclerotiorum* sRNAs (~*p* = 0.71, Fig. 5D). Conversely, during *S. sclerotiorum* infection, 68% of random *A. thaliana* gene samples had a lower ΔLFC than targets of *B. cinerea* sRNAs (~*p* = 0.68, Fig. 5E), whereas only 1% had a lower ΔLFC than targets of *S. sclerotiorum* sRNAs (~*p* = 0.01, Fig. 5F). Together, these analyses suggest that *S. sclerotiorum* sRNAs could have a negative impact on the expression of some *A. thaliana* genes during infection.

### Predicted targets of *S. sclerotiorum* sRNAs in *A. thaliana* are more likely to be associated with quantitative disease resistance

**Figure 6:**
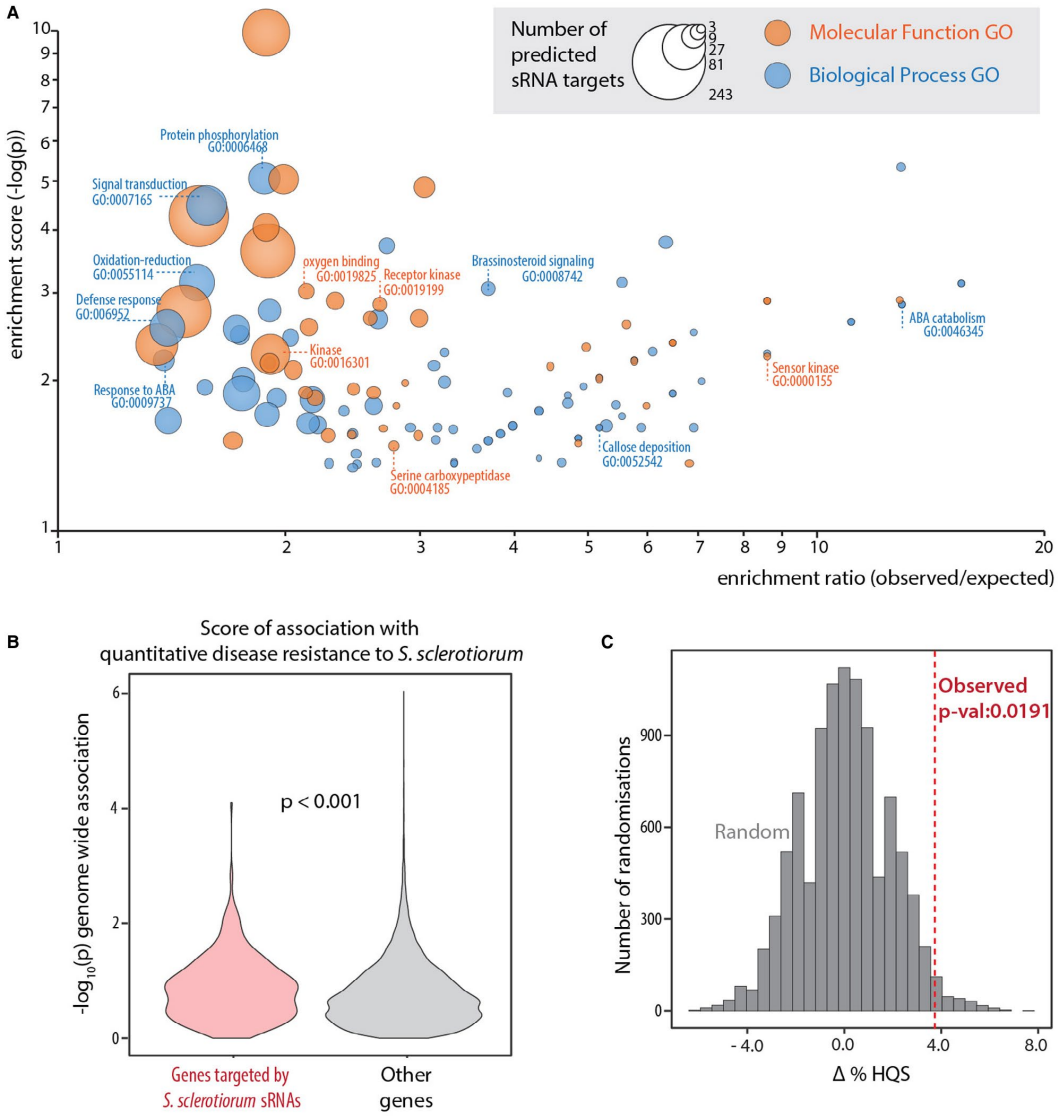
Predicted targets of *Sclerotinia sclerotiorum* sRNAs in *Arabidopsis thaliana* associated with quantitative disease resistance (QDR). (A) Enrichment (–log(*p*) (*y*‐axis)) of GO terms in putative *A. thaliana* targets of the 374 *S. sclerotiorum* core sRNAs. The ratio of the observed to expected proportions of the GO terms in sRNA targets is on the *x*‐axis. The size of points represents the number of predicted sRNA target candidates annotated with the GO term. Molecular function GO terms are in orange and biological process GO terms are in blue. Several terms discussed in the text are highlighted that indicate roles in signalling, hormone metabolism and defence against pathogens. (B) QDR scores (–log_10_(*P*) of association) (*y*‐axis) for *A. thaliana* genes predicted to be targeted by the 374 abundant *S. sclerotiorum* sRNAs (left) and other genes (right). *P* value for a Wilcoxon’s test is shown. (C) Distribution of ∆%HQS (difference in the % of genes with a QDR association score >1.3 between genes targeted or not by sRNAs) in 10 000 randomizations (grey). The vertical dashed line represents the observed ∆%HQS for *A. thaliana* genes targeted or not by *S. sclerotiorum* sRNAs.

We hypothesized that if predicted targets of *S. sclerotiorum* sRNAs in *A. thaliana* were involved in plant disease resistance, they may contain genetic markers associated with QDR against *S. sclerotiorum*. To support a role in disease resistance for predicted targets of *S. sclerotiorum* sRNAs in *A. thaliana*, we first performed gene ontology (GO) term and PFAM domain enrichment analyses using Fisher’s exact test. Among the top 1% gene functions enriched in sRNA targets (Fig. 6A, Data S3, Table S2), several were related to signalling (e.g. GO:0007165, GO:0019199, PF00069 and PF03107) and specifically plant immunity signalling (GO:0019199 and PF13855). Some annotations related to plant defence responses (GO:0006952, GO:0052542 and PF12662) were also in the top 2.5% enriched. Other annotations among the top 1% enriched for predicted *S. sclerotiorum* sRNA targets included ontologies and domains related to hormone metabolism (GO:0009737 and GO:0046345) and redox metabolism (GO:0055114, GO:0019825 and PF00175). Next, we exploited the probability of association with QDR against *S. sclerotiorum* for 204 648 single nucleotide polymorphisms (SNPs), obtained through a GWAS in 84 European accessions of *A. thaliana* (Badet *et al*., 2017a). We identified 122 034 SNPs distributed in 23 688 gene models, and determined a score of association per gene (–log_10_ of the *P* value for the most significant SNP for each gene). First, we found that the median score of association for predicted targets of *S. sclerotiorum* sRNAs was 0.82, significantly higher than the median score for non‐targets (median = 0.67, Wilcoxon’s rank sum test *P* = 4.872e^−05^, *W* = 4159300) (Fig. 6B). Genes with an association score >1.3 (corresponding to a *P* value < 0.05) represented 18.7% of *S. sclerotiorum* sRNA targets, but only 14.9% of other genes (difference in proportion of high QDR score Δ%HQS = 3.8, Fisher’s exact test *P* = 0.04). Second, we performed a randomization test to determine the likelihood of having a Δ%HQS ≥ 3.8 in random *A. thaliana* gene sets of the same size (Fig. 6C). We obtained a Δ%HQS < 3.8 in 98.1% of randomizations, indicating that *S. sclerotiorum* sRNA targets are significantly enriched in genes with an association score >1.3 (*P* = 0.0191).

### Mutants in *A. thaliana* kinase genes *SERK2* and *SNAK2* are more susceptible to *S. sclerotiorum* than wild‐type

**Figure 7:**
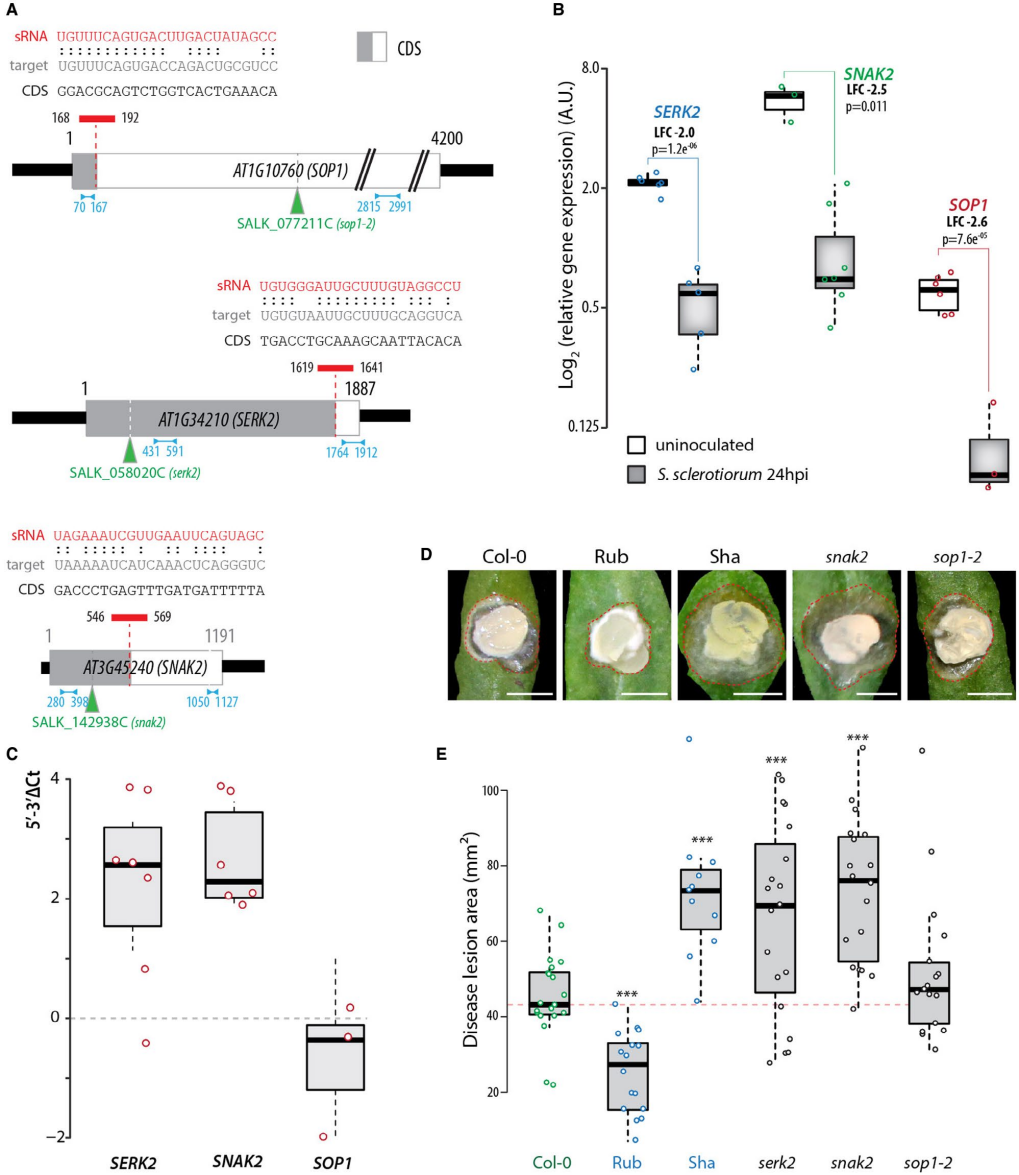
*SNAK2* and *SERK2* are two *Arabidopsis* genes associated with quantitative disease resistance identified based on *Sclerotinia sclerotiorum* sRNA analysis. (A) Schematic representation of the coding sequence from three top candidate sRNA targets showing the predicted sRNA target site. *Sclerotinia sclerotiorum* sRNA sequence is indicated in red, the corresponding predicted mRNA target sequence in grey and the corresponding CDS sequence in black. The position along the CDS is indicated relative to the transcription start site. Blue arrows indicate the position of amplicons generated in qRT‐PCR assays. Green triangles indicate the position of T‐DNAs in *A. thaliana* mutant lines used in this work. (B) Relative gene expression for three *A. thaliana* predicted targets of *S. sclerotiorum* sRNAs determined by qRT‐PCR in plants uninoculated and 24 hours post‐inoculation by *S. sclerotiorum*. Labels show average log_2_ (fold change) (LFC) on inoculation and the *P* value of a Welch *t*‐test. Values for three to seven independent biological samples are shown. (C) qRT‐PCR determination of 5′–3′Δ*C*_t_ as a reporter of mRNA stability in *A. thaliana* leaves inoculated by *S. sclerotiorum*. The 5′–3′Δ*C*_t_ corresponds to the difference in crossing time determined by qRT‐PCR for amplicons located at the 5′ and the 3′ ends of target mRNA. Values for 5′–3′Δ*C*_t_ increase when mRNA stability decreases. Boxplots show the first and third quartiles (box), median (horizontal line) and 90% confidence intervals (whiskers). Values were determined from three to seven distinct plants 24 hours post‐inoculation by *S. sclerotiorum*. (D) Representative disease symptoms 24 hours post‐inoculation by *S. sclerotiorum* on five *A. thaliana* genotypes. Red dotted lines delimit disease lesions. Bars: 0.5 cm. (E) *Arabidopsis* plants defective in *SERK2* and *SNAK2*, predicted targets of *S. sclerotiorum* sRNAs, were more susceptible to *S. sclerotiorum* than wild‐type plants. Lesion sizes were measured at 24 hours post‐inoculation on leaves from 11 to 20 plants. Similar results were obtained in two out of three independent biological experiments. *Arabidopsis* accessions Rubezhnoe (Rub) and Shahdara (Sha) were used as resistant and susceptible controls, respectively. Boxplots show the first and third quartiles (box), median (horizontal line) and the most dispersed values within 1.5 times the interquartile range (whiskers). Significance of the difference to Col‐0 was assessed by a Welch *t*‐test (***P < 0.001).

Among the top ten scores for association with QDR (Table 1), eight genes significantly down‐regulated *in planta* relative to *in vitro* (from –1.74 to –5.03 log_2_ fold). To rapidly test whether fungal sRNA analysis helps in identifying disease‐relevant genes, we searched for available homozygous mutant lines in genes from our top ten list. We identified SALK_077211C (*sop1‐2*) with a T‐DNA insertion in the 11th exon of *AT1G10760* encoding the α‐glucan, water dikinase SOP1/SEX1 required for starch degradation, SALK_142938C (*snak2*) with a T‐DNA insertion in the first intron of *AT3G45240* encoding the SNAK2 SNF1‐related kinase and SALK_058020C (*serk2*) with a T‐DNA insertion in the third intron of *AT1G34210* encoding the SERK2 somatic embryogenesis receptor‐like kinase 2 (Fig. 7A). To get support for the down‐regulation of the corresponding genes on *S. sclerotiorum* challenge, we performed quantitative reverse transcription‐polymerase chain reaction (qRT‐PCR) assays on wild‐type Col‐0 *A. thaliana* during *S. sclerotiorum* infection. We found an average log_2_ fold change of –2.6 for *SOP1* (Welch *t*‐test *P* value = 7.6e^−05^), –2.0 for *SERK1* (*P* = 1.2e^−06^) and –2.5 for *SNAK2* (*P* = 0.011) on inoculation with *S. sclerotiorum* (Fig. 7B; Table 1). Next, we measured 5′–3′Δ*C*_t_ for *SOP1*, *SERK2* and *SNAK2* as an estimate for RNA integrity (Vermeulen *et al.*, 2011). Using mRNAs from *S. sclerotiorum*‐infected samples, we obtained 5′–3′Δ*C*_t_ = –0.75 for *SOP1*, 2.2 for *SERK2* and 2.7 for *SNAK2* (Fig. 7C). The 90% confidence interval of 5′–3′Δ*C*_t_ spanned 0 for *SOP1* but not for *SERK2* and *SNAK2*, suggesting that *SOP1* is not a target of *S. sclerotiorum* sRNAs. High 5′–3′Δ*C*_t_ values for *SERK1* and *SNAK2* may result from targeting by *S. sclerotiorum* sRNAs, or from biases in the qRT‐PCR. We then measured the disease lesion area upon inoculation by *S. sclerotiorum* to evaluate QDR in these mutant lines (Fig. 7D,E). Twenty‐four hours after inoculation, disease lesions measured on average 45.5 mm^2^ in the Col‐0 wild‐type, 25.6 mm^2^ in the resistant accession Rubezhnoe (Welch *t*‐test *P* value = 0.0015) and 72.5 mm^2^ in the susceptible accession Shahdara (*P* value = 0.0021). Although mutants in *SOP1* are more susceptible to the fungal pathogen *Colletotrichum higginsianum* (Engelsdorf *et al.*, 2016), *sop1‐2* behaved similar to wild‐type upon inoculation by *S. sclerotiorum* in our assays (*P* value = 0.26). However, *snak2* and *serk2* mutant plants showed a ~30% increase in susceptibility to *S. sclerotiorum* compared to wild‐type (*P* value = 8.0e^−06^ and 0.0089, respectively). The kinetics of disease progression in these mutants suggest that they are impaired in the control of fungal colonization (Fig. S2). Based on our fungal sRNA analysis, we could identify *SERK2* and *SNAK2* as two *A. thaliana* genes likely involved in quantitative disease resistance to *S. sclerotiorum*. Further work will be required to determine the mechanisms through which *SERK2* and *SNAK2* contribute to resistance to *S. sclerotiorum* and whether *S. sclerotiorum* is able to alter their expression directly through sRNAs.

**Table 1:** Predicted target genes of *Sclerotinia sclerotiorum* sRNAs in *Arabidopsis thaliana* with the top 10 scores for association with quantitative disease resistance.

Gene id	Symbol	Description and annotation	LFC	Assoc. score
AT1G10760	GWD, GWD1, SEX1, SOP, SOP1, STARCH EXCESS 1	α‐glucan, water dikinase required for starch degradation. Involved in cold‐induced freezing tolerance. Mutations that eliminate the GWD protein or affect the dikinase domain of the enzyme dramatically reduce both the amount of phosphate in the amylopectin and the rate of starch degradation. Mature leaves of these mutants accumulate amounts of starch up to seven times greater than those in wild‐type leaves. Mutant more susceptible to *Colletotrichum higginsianum* penetration.	–5.0339	2.8870
			(*P* = 0)	
		PF01326 (PPDK_N); GO:0016310 phosphorylation; GO:0005524 ATP binding; GO:0016301 kinase activity	**–2.5890**	
			**(*P* = 7.6e^−05^)**	
AT1G22060		Sporulation‐specific protein	–3.4514	2.2089
		PF10358 (NT‐C2)	(*P* = 0)	
AT1G34210	ATSERK2, SERK2, SOMATIC EMBRYOGENESIS RECEPTOR‐LIKE KINASE 2	Plasma membrane LRR receptor‐like serine threonine kinase expressed during embryogenesis in locules until stage 6 anthers, with higher expression in the tapetal cell layer. SERK1 and SERK2 receptor kinases function redundantly as an important control point for sporophytic development controlling male gametophyte production. The mRNA is cell‐to‐cell mobile.	–2.2539	2.2100
		PF08263 (LRRNT_2); PF00069 (Pkinase); PF00560 (LRR_1); GO:0006468 protein phosphorylation; GO:0007165 signal transduction; GO:0004672 protein kinase activity; GO:0005102 signalling receptor binding; GO:0005515 protein binding	(*P* = 0)	
		GO:0005524 ATP binding	**–1.993**	
			**(*P* = 1.22e^−06^)**	
AT1G67120	ATMDN1, MDN1, MIDASIN 1	Homologue of the yeast MDN gene, which encodes a non‐ribosomal protein involved in the maturation and assembly of the 60S ribosomal subunit. In *Arabidopsis*, it is essential for female gametogenesis progression.	–2.6246	2.0966
		PF07728 (AAA_5); GO:0000027 ribosomal large subunit assembly; GO:0005524 ATP binding; GO:0016887 ATPase activity; GO:0005634 nucleus	(*P* = 0)	
AT2G13690		PRLI‐interacting factor	–4.6996	2.1121
		BTB/POZ domain‐containing protein (IPR038920)	(*P* = 0.0006)	
AT3G45240	ATSNAK2, GEMINIVIRUS REP INTERACTING KINASE 1, GRIK1	Geminivirus Rep interacting kinase. GRIKs are SnRK1 (SNF1‐related kinases) activating kinases. Both GRIKs specifically bind to the SnRK1 catalytic subunit and phosphorylate the equivalent threonine residue in its activation loop *in vitro*.	–0.0043	2.4200
			(*P* = 0.1558)	
		PF00069 (Pkinase); GO:0006468 protein phosphorylation; GO:0004672 protein kinase activity; GO:0005524 ATP binding	**–2.4805**	
			**(*P* = 0.01142)**	
AT3G49400		Transducin/WD40 repeat‐like superfamily protein	–2.9998	2.2120
		PF12657 (TFIIIC_delta); PF00400 (WD40); GO:0005515 protein binding	(*P* = 0.0002)	
AT4G26780	AR192, MGE2, MITOCHONDRIAL GRPE 2	Unknown function; thermotolerance to chronic heat stress in *Arabidopsis*	–4.4207	2.7703
		GrpE nucleotide exchange factor (IPR000740) PF01025 (GrpE); GO:0006457 protein folding; GO:0000774 adenyl‐nucleotide exchange factor activity; GO:0042803 protein homodimerization activity; GO:0051087 chaperone binding	(*P* = 0)	
AT4G28600	NO POLLEN GERMINATION RELATED 2, NPGR2	Calmodulin‐binding protein that is expressed in pollen, suspension culture cells, flowers and fruits	3.1327	4.1000
		PF13181 (TPR_8); PF14559 (TPR_19); GO:0005515 protein binding	(*P* = 0)	
AT4G36120		Filament‐like protein (DUF869)	–1.7418	2.3336
		Filament‐like plant protein (IPR008587)	(*P* = 0.0614)	

Log fold change and *P* values given in bold font were obtained by qRT‐PCR, values in regular font are from RNA‐seq.

## Discussion

In this study, we demonstrate that the broad host range plant pathogen *S. sclerotiorum* produces numerous abundant sRNAs expressed on two different hosts. These sRNAs exhibit characteristics typical of previously described filamentous pathogen sRNAs, such as a length distribution peaking between 20 and 25 nucleotides and a 5′ uridine bias (Mueth *et al.*, 2015; Vetukuri *et al.*, 2012; Yang, 2015). This echoes the findings of Zhou *et al.* (2012), who analysed sRNAs produced by *S. sclerotiorum in vitro*. The 5′ uridine has been shown to be important for sRNA functions in distantly related species, such as *Drosophila melanogaster* and *A. thaliana* (Ghildiyal *et al.*, 2010; Mi *et al.*, 2008). In these species, the presence of a 5′ uridine is important for directing the sRNAs to a specific Argonaut family protein, AGO1. This suggests that AGO1 (sscle03g027950) is a key player mediating RNA silencing in *S. sclerotiorum*. Although plant AGOs have undergone extensive diversification compared with animal AGOs, most canonical plant miRNAs are incorporated into AGO1 clade proteins (Fang and Qi, 2016). Thus, the 5′ uridine of sRNAs may be an important feature conserved in evolution to facilitate trans‐kingdom RNA silencing between plants and fungi. Here, we used 5′ U as a criterion to increase the specificity of our analysis pipeline. This may have filtered out fungal sRNAs relevant for infection. It is yet to be elucidated whether this bias in 5′ nucleotide exists among non‐plant pathogenic fungi.

Most of these sRNAs mapped to repetitive genomic regions, suggesting that sRNAs, like some fungal effectors, may be strongly associated with transposable elements. The repeat‐rich regions that contain effector genes often exhibit a reduced overall gene content. This phenomenon appears to be convergent among distantly related plant pathogens in both the fungal and oomycete classes (Croll and McDonald, 2012; Dong *et al.*, 2015; Raffaele and Kamoun, 2012; Rouxel *et al.*, 2011; Spanu *et al.*, 2010). In some species, effector genes within repeat‐rich regions exhibit high levels of sequence diversification and tend to evolve more rapidly than the rest of the genome, an observation encapsulated in the ‘two‐speed’ genome hypothesis (Dong *et al.*, 2015; Raffaele *et al.*, 2010; Seidl and Thomma, 2017). Clustering of repeats and effectors in specific genome niches probably results from selection against deleterious mutations in essential genes. Genome compartmentalization seems most pronounced in filamentous pathogens with a biotrophic or hemibiotrophic component to their lifestyles that specialize on one or a few host species (Raffaele *et al.*, 2010; Rouxel *et al.*, 2011; Spanu *et al.*, 2010). In contrast, broad host‐range necrotrophic fungal pathogens such as *S. sclerotiorum* and *B. cinerea* exhibit relatively repeat‐poor genomes (Derbyshire *et al.*, 2017; Van Kan *et al.*, 2017). Nevertheless, effector genes may also be associated with repeat sequences in necrotrophic fungi and fungi with a broad host range (Dallery *et al.*, 2017; Laurent *et al.*, 2017; Syme *et al.*, 2016; Wang *et al.*, 2017). Secretome analyses highlighted a number of candidate effector‐like proteins in the genome of *S. sclerotiorum* (Badet *et al.*, 2015; Derbyshire *et al.*, 2017; Guyon *et al.*, 2014; Heard *et al.*, 2015). These predicted effector genes associate with repeat sequences (Derbyshire *et al.*, 2017; Guyon *et al.*, 2014) and show a high degree of codon adaptation (Badet *et al*., 2017b). In this study, almost all sRNA loci were associated with transposable elements. Like effectors, *S. sclerotiorum* sRNA loci were, on average, further from gene sequences than genes were to each other. Although the number of polymorphisms in repetitive regions cannot be estimated with high accuracy, 10 kb windows including sRNA loci were clearly more polymorphic than the rest of the genome. These findings indicate that *S. sclerotiorum* exhibits a fast‐evolving, transposon‐associated sRNA effector repertoire. The fact that sRNA loci were highly polymorphic supports the hypothesis that these regions are an important component of adaptive evolution to the very diverse host environments that populations of broad host range fungi encounter in nature. It is possible to envisage a scenario under which random targeting of sRNAs to host immunity genes in one or more species confers a selective advantage. A rapid turnover of sRNAs and a large potential for inhibition of host genes through random sequence matches could thus create a high adaptive potential of *S. sclerotiorum* populations.

Many of the predicted host targets of the sRNAs identified in this study exhibited domains previously shown to function in plant immune responses (Kourelis and Van Der Hoorn, 2018). Furthermore, upon infection with *S. sclerotiorum*, these target genes were far more likely to be down‐regulated than non‐targets. The short sequences of sRNAs make the search for their targets prone to false positives. Our RNA integrity and mutant phenotype assays suggest that *SOP1* was mistakenly identified as a *S. sclerotiorum* sRNA target. A thorough analysis of the functionality of SOP1 in the *sop1.2* mutant, the analysis of more *SOP1* mutant alleles and complementary analyses of mRNA stability in cells infected by *S. sclerotiorum* will be required to rule out unambiguously SOP1 function in QDR against this fungus. Our analyses supported a role for *SERK2* and *SNAK2* in QDR against *S. sclerotiorum*. SERK2 has been associated with pathogen‐ and danger‐associated molecular pattern (PAMP and DAMP) signalling (Roux *et al.*, 2011; Yamada *et al.*, 2016). SERK2 also has a minor role in brassinosteroid (BR) signalling (Gou *et al.*, 2012), the overproduction of which confers enhanced resistance to *S. sclerotiorum* in *Brassica napus* (Sahni *et al.*, 2016). The reduced QDR of the *serk2* mutant to *S. sclerotiorum* may thus be explained by defects in PAMPs, DAMPs or BR signalling. SNAK2/GRIK1 phosphorylates and activates the sucrose non‐fermenting related kinase 1 (SnRK1) involved in energy and carbon signalling (Shen *et al.*, 2009). In wheat, SnRK1 interacts with TaFROG, a protein mediating host resistance to *Fusarium* head blight and the mycotoxin deoxynivalenol (Perochon *et al.*, 2015). In *Arabidopsis*, SnRK1 interacts with STOREKEEPER RELATED1/G‐Element Binding Protein (STKR1), which is associated with systemic acquired resistance and resistance to the oomycete *Hyaloperonospora arabidopsidis* (Nietzsche *et al.*, 2018). Similarly, deficiencies in the control of SnRK1 activity may underlie the enhanced susceptibility of the *snak2* mutant upon *S. sclerotiorum* inoculation. Although further functional assays will be required to draw firm conclusions, our data suggests that *S. sclerotiorum* may suppress host immunity genes with sRNAs.

To further understand the importance of trans‐kingdom RNA silencing during plant infection with *S. sclerotiorum* we tested the degree of association with quantitative disease resistance for the predicted sRNA targets in *A. thaliana* exploiting a previous GWAS (Badet *et al*., 2017a). The GWAS score was significantly higher in predicted *S. sclerotiorum* sRNA targets than non‐targets. In the *A. thaliana* GWAS, the *P* values of association with QDR remained, nevertheless, relatively modest for *S. sclerotiorum* sRNA targets (score ≤ 4.1, corresponding roughly to a false discovery rate of 1.0 × 10^−06^). Population structure, genetic and functional diversity in mapping populations, and rare and weak‐effect alleles can limit the ability of GWAS to detect causal loci in complex trait analyses (Bergelson and Roux, 2010). For instance, Corwin *et al.* (2016) used GWAS to associate 3504 *A. thaliana* genes with QDR to *B. cinerea*, including only 12 out of 101 genes previously demonstrated to contribute to *B. cinerea* resistance. Our meta‐analysis of the *P* values generated by the previous GWAS supports the hypothesis that predicted plant targets of sRNAs contain markers that are, on average, more strongly associated with a disease resistance phenotype based on a mixed linear model. These genes may not have been selected as relevant for QDR based on GWAS data alone. However, the combination of GWAS, RNA sequencing and fungal sRNA target predictions revealed novel candidate genes to be functionally characterized for association with QDR in the future. This combination of approaches may be useful in narrowing down candidate plant genes for functional characterization in diverse plant–pathogen interactions, particularly in the absence of high‐resolution genetic maps and for species in which functional tests are challenging.

## Experimental Procedures

### Fungal cultures and inoculation of host plants

*Sclerotinia sclerotiorum* isolate 1980 was grown on potato dextrose agar plates for 4 days at 24 °C. *Arabidopsis thaliana* accession Col‐0 and *P. vulgaris* genotype G19833 were grown for 4 weeks before inoculation, under controlled conditions at 22 °C, under a light intensity of 120 µmol/m^2^/s for 9 hours per day. Five‐millimetre wide plugs containing actively growing *S. sclerotiorum* mycelium were inoculated onto fully developed leaves. Inoculated plants were placed in Percival AR‐41 chambers at 80% humidity under the same day/light conditions as for plant growth for the duration of the infection. Samples for RNA extraction were harvested as described in Badet *et al.* (2017a), separating the centre and periphery of 2.5‐mm wide disease lesions with a scalpel blade. For the evaluation of QDR in *A. thaliana* SALK_077211C (*sop1*‐2), SALK_058020C (*serk2*) and SALK_142938C (*snak2*) 4‐week‐old plants were inoculated with *S. sclerotiorum* 1980 and lesion size analysed as described in Badet *et al*. (2017a). The experiment was repeated three times on 7 to 20 distinct plants per genotype.

### RNA extraction and sequencing

Small RNAs were extracted and sequenced as described in Badet *et al.* (2017b). Briefly, samples harvested *in planta* and from *S. sclerotiorum* grown *in vitro* in potato dextrose broth were processed using the NucleoSpin miRNA kit (Macherey‐Nagel, Düren, Germany) following the instructions of the manufacturer. Small RNA sequencing was performed by Fasteris SA (Geneva, Switzerland) on a HiSeq 2500 instrument using 50 bp single‐reads. The data is available at NCBI (accession SRP151049).

### Test for down‐regulated expression of fungal sRNA target genes in *A. thaliana*

To test whether predicted targets of fungal sRNAs in *A. thaliana* were more likely to be down‐regulated during infection, we assessed the LFC in the expression of these genes during fungal infection relative to controls. First, we compared the median LFC of all sRNA targets with non‐targets using a Wilcoxon test. Second, we used a non‐parametric randomization test based on the tests described in Hooton (1991). A randomization in our test consisted of assigning the status of ‘sRNA target’ and ‘non‐sRNA target’ to *A. thaliana* genes randomly and calculating the difference between mean LFC values (ΔLFC). For each randomization, we calculated ΔLFC as the mean LFC of non‐sRNA targets minus the mean LFC of sRNA targets. The *P* values obtained from the test were the number of times ΔLFC was lower than the actual mean ΔLFC difference between sRNA targets and non‐targets. We performed this analysis on four datasets: (1) predicted *A. thaliana* targets of *S. sclerotiorum* sRNAs identified in this study during infection with *S. sclerotiorum*, (2) predicted *A. thaliana* targets of *B. cinerea* sRNAs identified in Weiberg *et al.* (2013) during infection with *S. sclerotiorum*, (3) predicted *A. thaliana* targets of *S. sclerotiorum* sRNAs during infection with *B. cinerea* and (4) predicted *A. thaliana* targets of *B. cinerea* sRNAs during infection with *B. cinerea*. For quantitative RT‐PCR, assays were performed as described in Badet *et al.* (2019), using *AT2G28390* as reference housekeeping gene. Oligonucleotide primers used are given in Table S3.

### Analysis of the genomic context of sRNA loci

Two analyses of the genomic context of sRNAs were performed. First, all loci where sRNAs mapped, including multiple mappings, were considered. The positions of these were calculated with respect to repeat annotations from Derbyshire *et al. *(2017) using the Bedtools module ‘intersect’ with the option ‘‐d’. Then, the distances of each of these to the nearest 5′ and 3′ genes were calculated using a Python script. The same was done for each gene to the nearest neighbouring gene from the 5′ or 3′ end. These distances were compared between genes and sRNA mapping sites using a Wilcoxon’s test. Distances for genes that were either predicted effectors (Derbyshire *et al.*, 2017) or not predicted effectors were also compared using a Wilcoxon’s test.

To assess how polymorphic sRNA loci were, SNP content was assessed based on a panel of 25 Illumina‐sequenced *S. sclerotiorum* isolates aligned to the 1980 reference genome. These isolates were derived from Derbyshire *et al. *(2019) and we followed the same mapping and variant calling procedures described in this publication. Importantly, we included a depth filter to remove repeat‐induced read mappings. The genome was split into 10 kb sliding windows using Bedtools and the number of overlaps with SNPs in VCF format for each window were calculated with the Bedtools module ‘intersect’ with the option ‘‐c’. The windows were then divided into those containing sRNA mapping loci and those not containing them and the number of SNPs for each type of window was compared using a Wilcoxon’s test. Similar analyses were conducted for sRNA loci predicted by the software package ‘CoLIde’ (Mohorianu *et al.*, 2013). To assess distance to the nearest neighbouring gene we used the Bedtools module ‘closest’. We either counted genes overlapping the sRNA loci (+OLGs) and gave them a value of 0 for distance or considered the nearest non‐overlapping gene (–OLGs). We compared these distances with the distance between each gene and the nearest neighbouring gene using a randomization test (described in detail under ‘Test for down‐regulated expression of fungal sRNA target genes in *A. thaliana*’). We performed the same test as above for the number of polymorphisms in sliding windows containing CoLIde sRNA loci vs the number in sliding windows not containing CoLIde sRNA loci.

### Quality filtering and mapping of sRNA reads

Reads were quality filtered using Trimmomatic v. 0.22 (Bolger *et al.*, 2014) with the settings ‘ILLUMINACLIP:Adapters.fasta:2:1:1’, where ‘Adapters.fasta’ is a file containing adapter sequences, and their reverse complements, for the Illumina small RNA library prep kit. After quality filtering, reads from all samples were subjected to a further alignment‐based filtering procedure. Bowtie v. 1 with the settings ‘‐v 0’ and ‘‐a’, to obtain all exact genomic matches, was used for each step. For *A. thaliana*, reads from all samples that mapped to the sense strand of reads in the mock sample were discarded (antisense alignments were not discarded as small RNA sequencing is stranded). Then, reads that mapped to the sense strand of host gene transcripts, host gene non‐coding RNAs downloaded from Rfam (v. 13.0) and *S. sclerotiorum* non‐coding RNAs downloaded from Rfam were discarded sequentially. Only reads that passed all of these steps and that were subsequently exactly mapped to the reference genome of *S. sclerotiorum* 1980 (Derbyshire *et al.*, 2017) were kept. The same procedure was followed for *P. vulgaris* using sRNA sequences predicted for this species obtained by Formey *et al.* (2015). For differential expression analysis, the two filtering procedures for the two different hosts were both applied to the *in vitro* sample to create two different filtered *in vitro* datasets for analysis. This controlled for differences in filtering procedures that may cause artificial observation of changes in sRNA expression. To determine whether sRNAs were likely derived from higher confidence sRNA biogenesis loci, we used the software module CoLIde (Mohorianu *et al.*, 2013).

### Differential expression analyses of small RNAs

Differential expression analysis was performed using DESeq v. 1.22.11 (Anders and Huber, 2010). Each unique sRNA was considered a single entity with a raw read count in this analysis. All *in planta* samples were compared with *in vitro* samples using a negative binomial test. Unique reads that exhibited a *P* adjusted value of below 0.05 were considered differentially expressed.

### Small RNA target prediction

To predict targets of *S. sclerotiorum* sRNAs, we used the psRNATarget online server (Dai and Zhao, 2011). For the over‐representation test of GO terms in *A. thaliana,* we used an *E* value of ≤ 3, resulting in 1368 predicted sRNA target genes (1789 including alternately spliced transcripts). For all other tests, we used a more stringent *E* value of ≤ 2.5, resulting in 408 predicted sRNA target genes (539 including alternately spliced transcripts). We used the same technique to predict targets of *B. cinerea* sRNAs in the *A. thaliana* genome. All other parameters were default. For all species in which we predicted sRNA targets, we first compared protein sequences against RepBase v. 23.05 (Jurka *et al.*, 2005) using BLASTp. Any mRNAs whose proteins were homologous to sequences in RepBase with an *E* value of ≤ 1.0e^−10^ were not used in target prediction. This is because these proteins are likely host transposable element genes, which could be similar to fungal sRNAs by virtue of their shared evolutionary origins.

### Test for over‐representation of functional domains

To test for over‐representation of GO terms among *A. thaliana* predicted targets of *S. sclerotiorum* sRNAs, we used the R bioconductor package TopGO (Alexa *et al.*, 2006). We performed the test separately for the molecular function and biological process categories of GO terms. In each instance, we used terms with five or more annotated genes. *P* values for GO and PFAM enrichment were obtained using Fisher’s exact test and adjusted with the Benjamini–Hochberg correction (Benjamini and Hochberg, 1995).

### Messenger RNA sequencing and differential expression analysis

The expression of *A. thaliana* Col‐0 genes during *S. sclerotiorum* infection corresponds to expression at the edge of necrotic lesions relative to non‐challenged plants, as reported in Badet *et al.* (2017a), data downloaded from the NCBI Gene Expression Omnibus (accession GSE106811). To determine expression of *A. thaliana* genes during *B. cinerea* infection, we obtained a dataset from Coolen *et al.* (2016) from the NCBI sequence read archive (https://www.ncbi.nlm.nih.gov/sra). The accessions were SRX1705130, SRX1705129 and SRX1705128, mock‐inoculated plants and SRX1728, SRX1729, SRX1730, 24 hours post‐inoculation with *B. cinerea*. DESeq2 v. 1.10.1 was used to assess LFC between the mock‐inoculated and *B. cinerea-*infected samples.

### Test for over‐representation of significant quantitative disease resistance scores among sRNA targets

To determine whether targets of *S. sclerotiorum* sRNAs were more likely to be significantly associated with QDR, we used the previously assessed markers from Badet *et al*. (2017a). We considered only genes that contained SNP markers, and only the most significant (highest –log_10_(*P*)) SNP in each gene. First, we conducted a Wilcoxon rank sum test of difference in median –log_10_(*P*). Second, we used a similar randomization test to the test used for down‐regulated expression of sRNA targets. In this instance, each randomization recorded the proportion of targets and non‐targets that exhibited a –log_10_(*P*) value of above 1.3 (or an untransformed score of below 0.05). The score for each randomization was the proportion of targets with a significant SNP minus the proportion of non‐targets with a significant SNP. This was termed the Δ%HQS. The *P* value derived from this test was the proportion of randomizations with Δ%HQS higher than the actual data. Third, we performed a Fisher’s exact test of over‐representation to test whether genes with significant SNPs were over‐represented among genes that were predicted *S. sclerotiorum* sRNA targets.

## Supporting information


**Fig. S1** Genomic context of sRNA loci predicted by CoLIde. (A) The results of a randomization test of distance to nearest gene for all genes minus distance to nearest gene for sRNA loci. The *x*‐axis shows this difference and the *y*‐axis shows the frequency at which it was observed in the 1000 randomizations. Top panel: for sRNA loci overlapping genes, the nearest non‐overlapping gene is used (–OLGs) (*P* = 0). Bottom panel: for sRNA loci overlapping genes, the overlapping gene is counted and given a distance of zero (+OLGs) (*P* = 0.001). (B) A violin plot of the distribution of distances to nearest neighbouring genes for genes, CoLIde loci (sRNAs) not including overlapping genes (–OLGs) and including overlapping genes (+OLGs). The *y*‐axis shows the distance to nearest neighbouring gene for genes and for sRNAs + OLGs includes zero values for overlapping genes. (C) A violin plot of the number of SNPs in 10 kb windows that contain (+sRNAs) or do not contain (–sRNAs) CoLIde sRNA loci (*P* = 2.2e^‐16^). The *y*‐axis shows the number of SNPs detected among a panel of 25 *Sclerotinia* *sclerotiorum *isolates.

**Fig. S2** Complementary information on the disease resistance phenotype of plants mutated in predicted targets of *Sclerotinia sclerotiorum* sRNAs. (A) Lesion areas measured 24 hours post‐inoculation by *S. sclerotiorum* (mm²) in three independent biological experiments. For each experiment, leaves from *n* = 5 to *n* = 20 distinct plants were analysed. The *P* value for a Welch two‐sample *t*‐test comparing each genotype to Col‐0 wild‐type is indicated. Experiment 2 showed unusually slow disease progression and high variability on Col‐0, preventing the detection of differences between genotypes. (B) Progression of disease lesions between 1000 and 2000 min post‐inoculation in wild‐type and mutant lines (12 plants per genotype). Dots and lines show mean values for each genotype, error bars show standard errors of the mean. (C) Disease lesion area at 24 hours post‐inoculation extracted from the data shown in (B). (D) Time to first lesion appearance estimated as the time when lesions reached 400 pixels. This parameter was not differential between plant genotypes. (E) Lesion growth speed (in pixel/min) between 1300 and 1500 min post‐inoculation. The *P* values of Welch two‐sample *t*‐tests comparing each genotype to Col‐0 wild‐type are indicated in (C)–(E). Boxplots show the first and third quartiles (box), median (thick line) and the most dispersed values within 1.5 times the interquartile range (whiskers).

**Table S1** Size distribution (in nucleotides) of library fragments selected for Illumina sequencing (% total fragments).

**Table S2** Enrichment of GO terms in *Arabidopsis thaliana* gene targets of 374 *Sclerotinia sclerotiorum* core sRNAs at a psRNATarget *E* value of ≤3.

**Table S3** List of oligonucleotide primers used in this work.

**Data S1** Nucleotide sequence of the 374 abundant sRNA from *Sclerotinia sclerotiorum*.

**Data S2** Mapping positions for 374 abundant sRNAs in the genome of *Sclerotinia sclerotiorum*.

**Data S3** Predicted targets in *Arabidopsis thaliana* genome for 374 abundant sRNAs produced by *Sclerotinia sclerotiorum*.
