# Supplementary figures and images for: Small RNAs from the plant pathogenic fungus Sclerotinia sclerotiorum highlight host candidate genes associated with quantitative disease resistance
Source: Mol Plant Pathol. 2019 Jul 30;20(9):1279–97. doi: 10.1111/mpp.12841 (PMC6715603; doi:10.1111/mpp.12841)

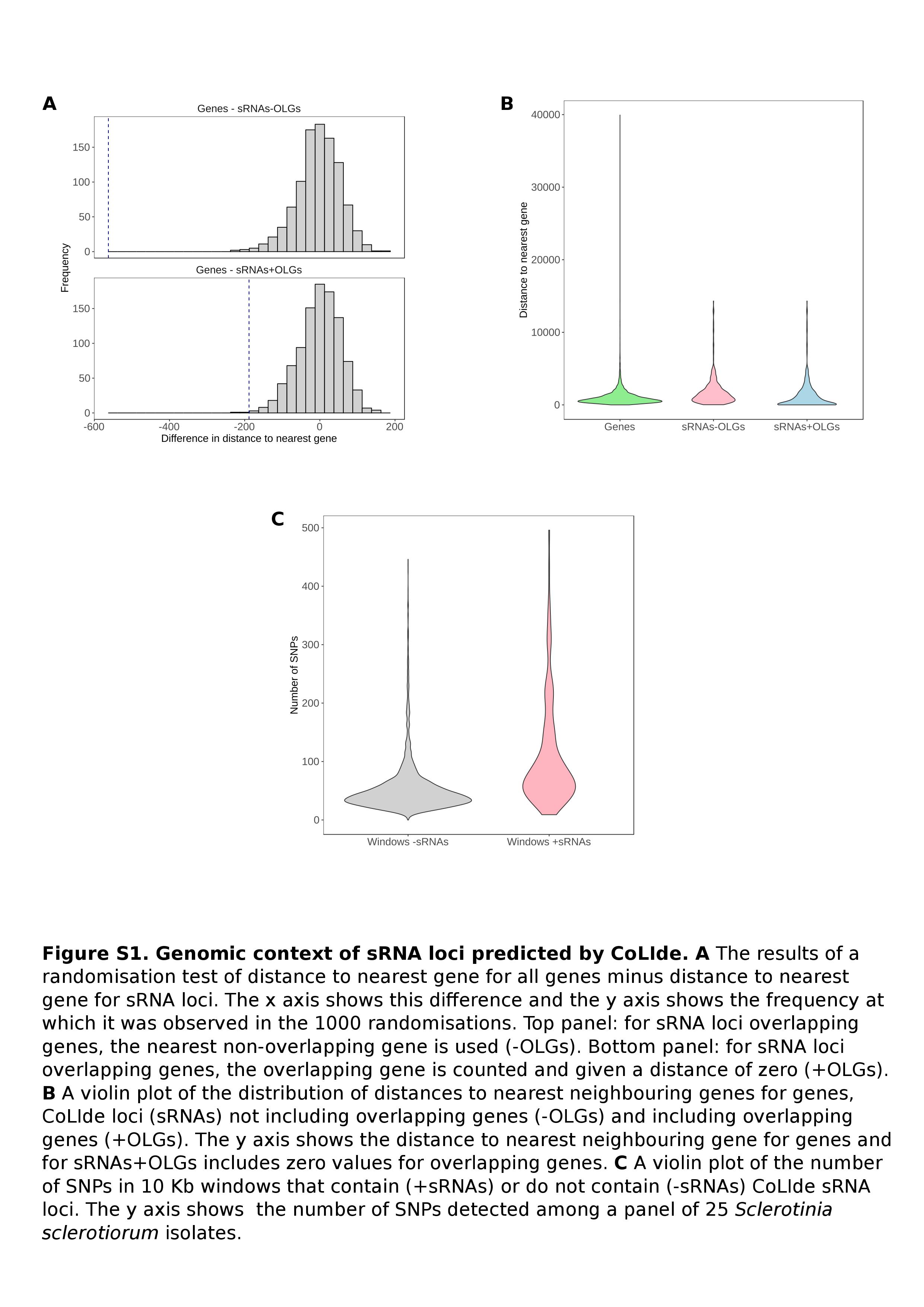

Supplement: Supplementary file 1 — Fig. S1 Genomic context of sRNA loci predicted by CoLIde. (A) The results of a randomization test of distance to nearest gene for all genes minus distance to nearest gene for sRNA loci. The x‐axis shows this difference and the y‐axis shows the frequency at which it was observed in the 1000 randomizations. Top panel: for sRNA loci overlapping genes, the nearest non‐overlapping gene is used (–OLGs) (P = 0). Bottom panel: for sRNA loci overlapping genes, the overlapping gene is counted and given a distance of zero (+OLGs) (P = 0.001). (B) A violin plot of the distribution of distances to nearest neighbouring genes for genes, CoLIde loci (sRNAs) not including overlapping genes (–OLGs) and including overlapping genes (+OLGs). The y‐axis shows the distance to nearest neighbouring gene for genes and for sRNAs + OLGs includes zero values for overlapping genes. (C) A violin plot of the number of SNPs in 10 kb windows that contain (+sRNAs) or do not contain (–sRNAs) CoLIde sRNA loci (P = 2.2e‐16). The y‐axis shows the number of SNPs detected among a panel of 25 Sclerotinia sclerotiorum isolates. [file MPP-20-1279-s001.jpg]

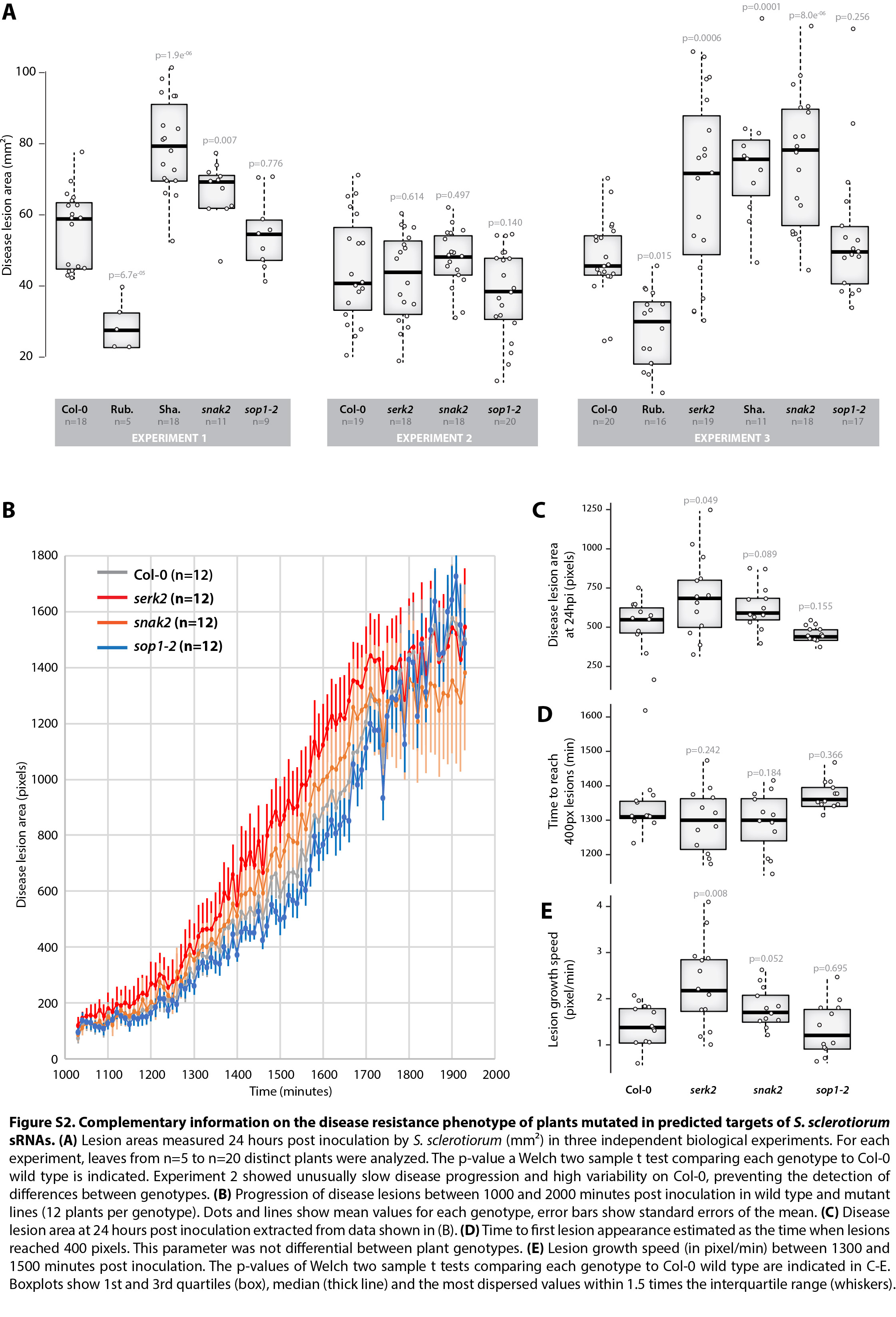

Supplement: Supplementary file 2 — Fig. S2 Complementary information on the disease resistance phenotype of plants mutated in predicted targets of Sclerotinia sclerotiorum sRNAs. (A) Lesion areas measured 24 hours post‐inoculation by S. sclerotiorum (mm²) in three independent biological experiments. For each experiment, leaves from n = 5 to n = 20 distinct plants were analysed. The P value for a Welch two‐sample t‐test comparing each genotype to Col‐0 wild‐type is indicated. Experiment 2 showed unusually slow disease progression and high variability on Col‐0, preventing the detection of differences between genotypes. (B) Progression of disease lesions between 1000 and 2000 min post‐inoculation in wild‐type and mutant lines (12 plants per genotype). Dots and lines show mean values for each genotype, error bars show standard errors of the mean. (C) Disease lesion area at 24 hours post‐inoculation extracted from the data shown in (B). (D) Time to first lesion appearance estimated as the time when lesions reached 400 pixels. This parameter was not differential between plant genotypes. (E) Lesion growth speed (in pixel/min) between 1300 and 1500 min post‐inoculation. The P values of Welch two‐sample t‐tests comparing each genotype to Col‐0 wild‐type are indicated in (C)–(E). Boxplots show the first and third quartiles (box), median (thick line) and the most dispersed values within 1.5 times the interquartile range (whiskers). [file MPP-20-1279-s002.jpg]
